# Targeted genomic CRISPR-Cas9 screen identifies MAP4K4 as essential for glioblastoma invasion

**DOI:** 10.1038/s41598-019-50160-w

**Published:** 2019-09-30

**Authors:** Laura M. Prolo, Amy Li, Scott F. Owen, Jonathon J. Parker, Kara Foshay, Ryan T. Nitta, David W. Morgens, Sara Bolin, Christy M. Wilson, Johana C. M. Vega L, Emily J. Luo, Gigi Nwagbo, Allen Waziri, Gordon Li, Richard J. Reimer, Michael C. Bassik, Gerald A. Grant

**Affiliations:** 1 Stanford University School of Medicine, Department of Neurosurgery, 300 Pasteur Dr, Stanford, CA 94305 USA; 2 Stanford University School of Medicine, Department of Genetics, 291 Campus Dr, Stanford, CA 94305 USA; 3 J. David Gladstone Institute of Neurological Disease, 1650 Owens St, San Francisco, CA 94158 USA; 4 Inova Neuroscience and Spine Institute, Inova Health Systems, 8110 Gatehouse Rd, Falls Church, VA 22042 USA; 5 Stanford University School of Medicine, Department of Neurology, 300 Pasteur Dr, Stanford, CA 94305 USA

**Keywords:** CNS cancer, Cancer in the nervous system

## Abstract

Among high-grade brain tumors, glioblastoma is particularly difficult to treat, in part due to its highly infiltrative nature which contributes to the malignant phenotype and high mortality in patients. In order to better understand the signaling pathways underlying glioblastoma invasion, we performed the first large-scale CRISPR-Cas9 loss of function screen specifically designed to identify genes that facilitate cell invasion. We tested 4,574 genes predicted to be involved in trafficking and motility. Using a transwell invasion assay, we discovered 33 genes essential for invasion. Of the 11 genes we selected for secondary testing using a wound healing assay, 6 demonstrated a significant decrease in migration. The strongest regulator of invasion was mitogen-activated protein kinase 4 (MAP4K4). Targeting of MAP4K4 with single guide RNAs or a MAP4K4 inhibitor reduced migration and invasion *in vitro*. This effect was consistent across three additional patient derived glioblastoma cell lines. Analysis of epithelial-mesenchymal transition markers in U138 cells with lack or inhibition of MAP4K4 demonstrated protein expression consistent with a non-invasive state. Importantly, MAP4K4 inhibition limited migration in a subset of human glioma organotypic slice cultures. Our results identify MAP4K4 as a novel potential therapeutic target to limit glioblastoma invasion.

## Introduction

Glioblastoma is the most common malignant primary brain tumor in adults and carries a poor prognosis with limited therapeutic options. Invasion of glioblastoma cells into normal brain parenchyma renders malignant cells inaccessible to surgical intervention and poised to drive tumor recurrence. Furthermore, it has been suggested that the migratory tumor cells are more resistant to apoptosis, thus making chemotherapeutic agents and radiation less effective^1–3^.

Cell migration is a fundamental process that is necessary for development and wound repair, and is co-opted by cancerous cells to allow invasion. Tumor cell invasion is a complex process that is driven by a combination of cytoskeletal rearrangements, signaling by cell adhesion receptors, and secretion of matrix-modifying proteases. Tumor cells interact extensively with the extracellular matrix and with normal cells of the central nervous system through each of these mechanisms^4,5^, and inhibition of any of these processes has the potential to prevent an invasive phenotype. Understanding the molecular mechanisms and signaling pathways that drive migration and invasion in glioblastoma may not only lead to better biomarkers of tumor status but also lead to novel therapeutic targets.

We performed the first large-scale CRISPR-Cas9 screen in glioblastoma specifically designed to identify novel genes essential for invasion^6–11^. Using a pooled library approach, we systematically evaluated the effect of loss of individual gene function on a targeted genomic scale. Lentivirally delivered sgRNAs were used to produce a stable loss of function library in the human glioma U138 line and separation of invasive phenotypes was performed using a transwell invasion assay. Using next generation sequencing we identified genes essential for glioblastoma survival as well as directional invasion. The gene with the most significant effect in this novel screen was MAP4K4 (mitogen-activated protein 4 kinase 4). To establish the importance of MAP4K4 function in cell motility for glioblastoma beyond the U138 human glioma line used for this screen, we used a specific drug inhibitor to demonstrate reduced migration in a wound healing assay in three additional adult malignant glioma lines. Using this drug in a human tumor organotypic slice culture limited migration in a subset of gliomas. Furthermore, we found that loss of MAP4K4 function drives glioblastoma cells into a non-invasive state. In summary, we used CRISPR-Cas9 to screen for genes essential for human glioblastoma invasion and found that MAP4K4 plays an important role in brain tumor invasion. MAP4K4 may therefore become an important therapeutic target.

## Results

### High-throughput CRISPR screen for glioblastoma invasion

**Figure 1:**
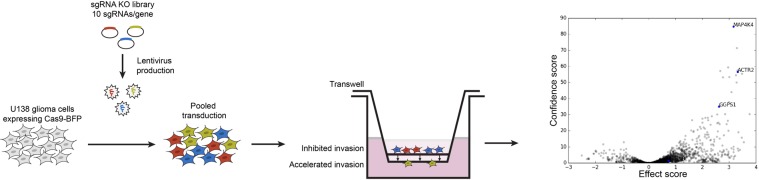
Pooled sgRNA CRISPR-Cas9 screening approach. The U138-Cas9-BFP human glioma line was infected with lentiviral sgRNA libraries targeting cell motility and potential drug targets. The infected cells were expanded and seeded onto matrigel coated transwell inserts and allowed to invade toward a higher concentration of serum for 36 hours. Cells remaining in the upper chamber (blue and red, inhibited invasion) and cells in the lower chamber (yellow, accelerated invasion) were collected. sgRNAs were PCR amplified from genomic DNA and subjected to Illumina sequencing. A volcano plot for all genes targeted in the screen shows genes enriched in the bottom chamber (gray dots to the left of midline) and the top chamber (gray dots to the right of midline). Blue dots demonstrate three examples of genes enriched in the non-invasive population. No genes were statistically enriched in the invasive population.

To identify novel genes that are essential for glioblastoma invasion, we performed a CRISPR-Cas9 screen using a pooled approach (Fig. 1). First, we generated a stable U138 cell line expressing Cas9 fused to blue florescent protein (BFP). These transduced cells were subsequently infected with lentiviral sgRNA libraries targeting 4,574 genes which were predicted to be involved with cell motility or serve as drug targets (sub-libraries used (1) drug targets, kinases phosphatases and (2) trafficking, mitochondrial, motility)^12^. The libraries also contained an additional 2,943 sgRNA negative controls targeting non-functional, non-genic regions as well as non-targeting controls. Each plasmid in the library contained mCherry and a sgRNA targeting a single gene. Overall the library consisted of 10 sgRNAs per gene and we maintained the infected cell number at 1000X representation of the number of library elements. We targeted a low infection efficiency (MOI 0.2) to decrease the chances of a cell being infected by more than one sgRNA. The library-infected pool of cells was then treated with puromycin for seven days to remove uninfected cells and achieve a population of cells that was ~90% positive for mCherry. The U138 Cas9 library was expanded, seeded onto the center of a transwell with matrigel and allowed to invade toward a higher concentration of serum in the bottom chamber for 36 hours. At this time point approximately 6% of cells had invaded. The population of cells in the upper and lower chambers were collected separately and genomically integrated sgRNAs were extracted, amplified by PCR, and analyzed using deep sequencing. We used a 10% False Discovery Rate as a cutoff to identify significant gene hits^13^. Ninety-eight gene targets were significantly disenriched from the expanded, starting library, identifying genes necessary for glioblastoma survival (Supplementary Table 1). Thirty-three gene targets were enriched in the non-migrating population of cells, suggesting that they are essential for invasion (Supplementary Table 2). This population included expected hits such as PDGFRα, which has previously been shown to be necessary for glioblastoma invasion, suggesting our assay was capable of identifying known regulators of migration^14,15^. We chose to pursue 11 novel gene hits based on having a significant migration effect without a survival effect.

### Validation of screening results using wound healing assay

**Figure 2:**
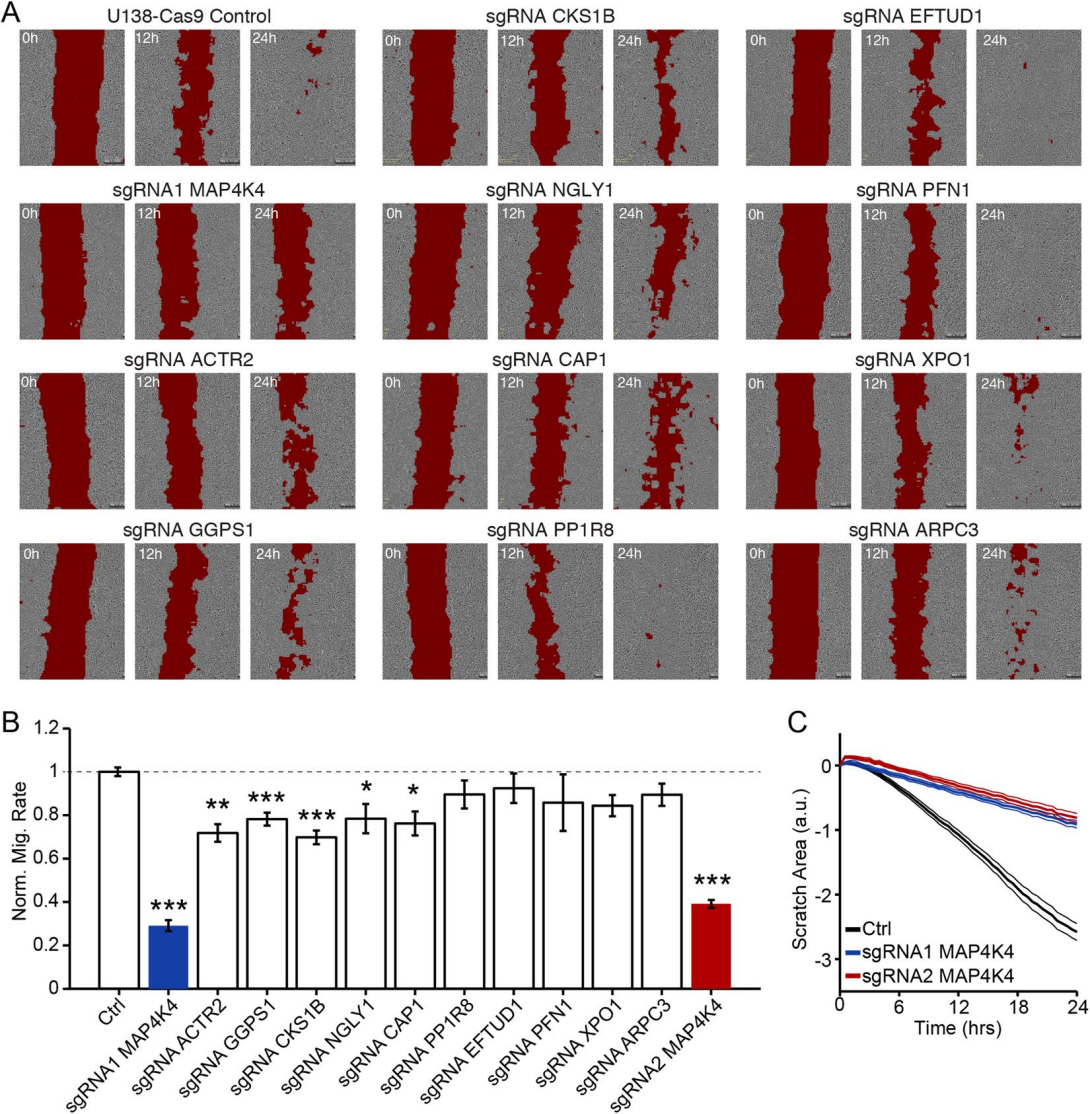
Validation of genes inhibiting migration using a wound healing assay. Eleven genes significantly enriched in the noninvasive population from the screen were validated in a wound healing assay. (**A**) Scratch area (red) is identified at 0, 12 and 24 hours in a representative monolayer of the specified cell line. (**B**) Quantification of normalized migration rate of each cell line calculated from 6–12 hours, n ≥ 7 for each condition, *p < 0.05, **p < 0.01, ***p < 0.001. (**C**) Average scratch area over time for the control U138-Cas9 cell line (solid black line +/− SD) and MAP4K4 knockout lines using two separate sgRNA sequences (red and blue lines +/− SD).

In order to validate gene hits from our screen, we made individual U138 Cas9 cell lines for 11 of the top gene hits. Each cell line was transduced with a unique sgRNA that was identified in the screen as having the greatest effect against migration out of the 10 sgRNAs per gene tested. Specifically, we chose kinases (MAP4K4, CKS1B), regulators of actin polymerization (ACTR2, ARPC3, PFN1, CAP1), and genes that have not been thoroughly characterized (GGPS1, NGLY1, PPP1R8, EFTUD1, XPO1). We chose to use a wound-healing assay to test whether these genes are essential for migration in general, and to ensure that the impaired migration phenotype was not specific to the transwell assay. Cells double positive for both Cas9-BFP and the sgRNA-GFP targeting the specified genes above were plated at a density of 75,000/well in a 24 well plate. A line of cells was removed through the confluent monolayer using a pipet tip and images were collected every 30 to 60 min for at least 24 h using an IncuCyte live cell analysis system (Fig. 2). To calculate the rate of scratch closure accurately and quantitatively, we developed a Matlab script that calculated scratch area based on local variation in image brightness (red fill, Fig. 2A). Six of the 11 genes we targeted led to a significant reduction in rate of migration (Fig. 2B). The gene with the greatest effect on both the transwell and wound healing assays was MAP4K4 (Fig. 2C). To confirm the effect on migration was not due to disruption of an off-target gene, we created a second MAP4K4 knock-out line using a different sgRNA. The second knock-out demonstrated an equally significant deficiency in migration (Fig. 2C). Notably targeting ACTR2, which encodes a direct phosphorylation substrate for MAP4K4, also significantly decreased migration (Fig. 2A,B).

### Inhibiting MAP4K4 blocks migration of four adult glioblastoma cell lines

**Figure 3:**
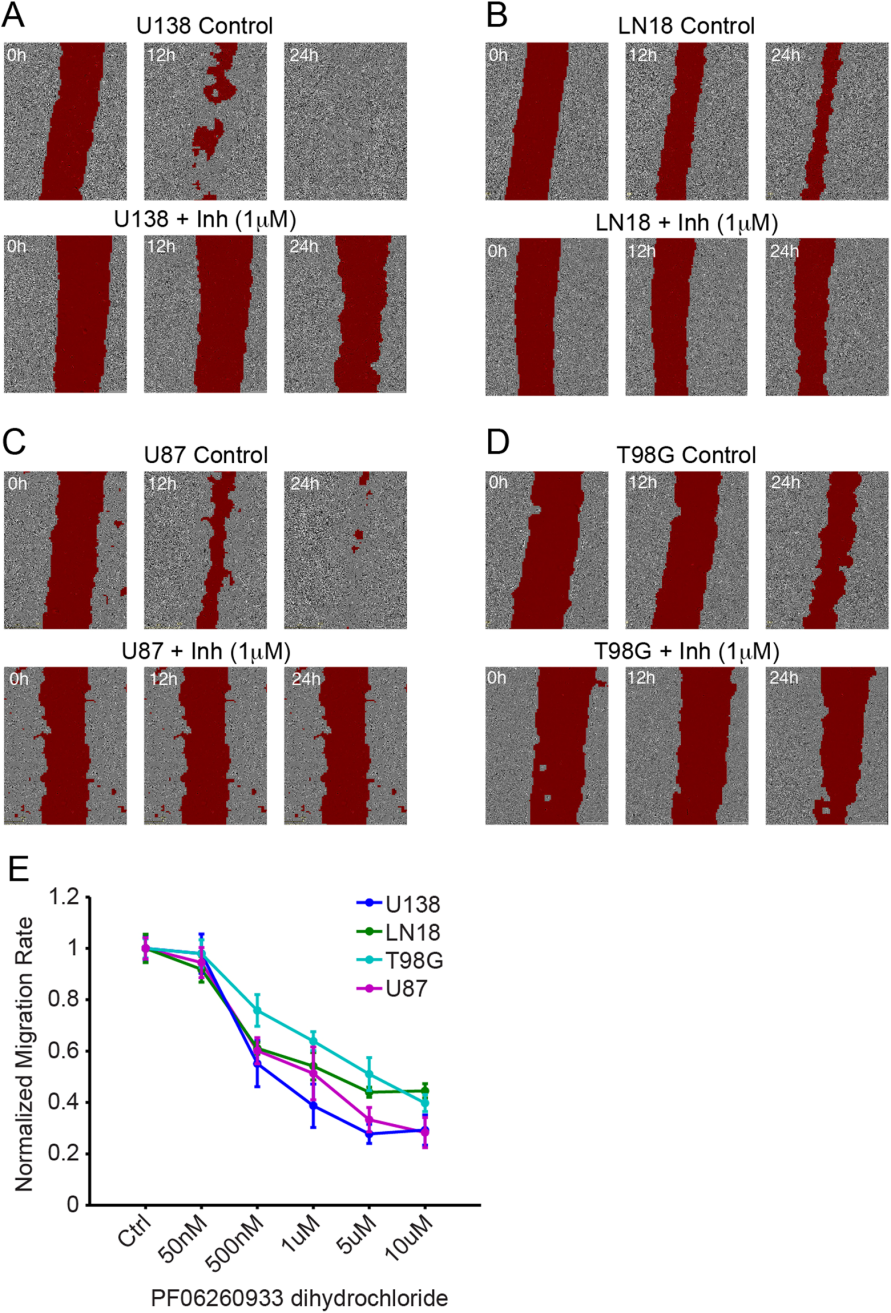
Inhibiting MAP4K4 slows migration in multiple glioma lines. The MAP4K4 inhibitor, PF06260933 dihydrochloride, slows migration in a wound healing. Scratch area (red) is marked at the specified time points in a representative monolayer of the identified cell lines: (**A**) U138, (**B**) LN18, (**C**) U87-MG, (**D**) T98G. For each cell line top row is representative control and bottom row is representative 1 μM drug treatment. (**E**) Quantification of normalized migration rate of each cell line calculated at 6–12 hours, n ≥ 4 for each concentration.

To assess whether our results were specific to the U138 genetic background or whether MAP4K4 acts as a more general regulator of glioblastoma migration we tested the effect of a small-molecule inhibitor of MAP4K4 (PF 06260933 dihydrochloride) on migration in three additional adult glioma lines (LN18, U87-MG, T98G). Using the wound healing assay we found a dose dependent response in scratch closure in all cell lines tested (Fig. 3). These findings suggest MAP4K4 is a broad regulator of glioblastoma migration and not specific to a particular genetic background. To assess whether this effect was simply due to cell death rather than decreased migration we performed a cell viability assay looking at metabolic activity of cells by measuring their ability to convert resazurin into fluorescent resorufin. U138 cell viability remained above 90% in the presence of PF06260933 dihydrochloride demonstrating the drug does not affect viability of human glioma cells (Supplementary Fig. 1A). We also assessed viability over 48 hours in control and MAP4K4 knock-out lines and found no statistical difference between the two lines suggesting similar growth rates and viability (Supplementary Fig. 1B).

### MAP4K4 is essential for invasion of U138 cells

**Figure 4:**
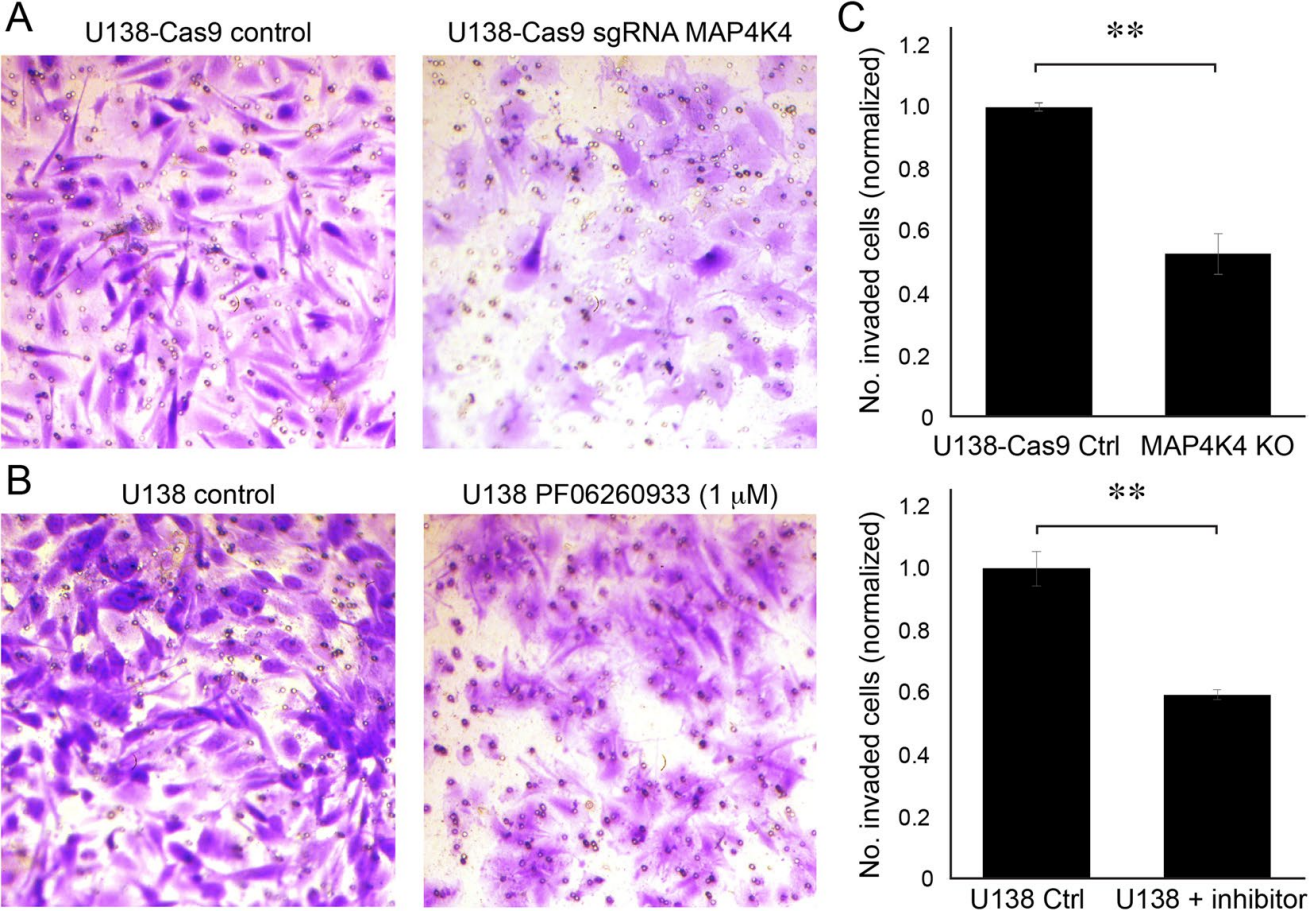
Knocking out or inhibiting MAP4K4 reduces invasion in the transwell invasion assay. (**A**) Representative area of the lower membrane surface of a matrigel invasion chamber showing invaded U138 cells stained with cresyl violet. (**A**) U138-Cas9 control (left) compared to MAP4K4−/− (right). (**B**) U138 control compared to U138 cells treated with 1 μM of MAP4K4 inhibitor (PF06260933 dihydrochloride). (**C**) Quantification of cell invasion with cell counts normalized to invasion of controls, n = 3, **p < 0.01.

**Figure 5:**
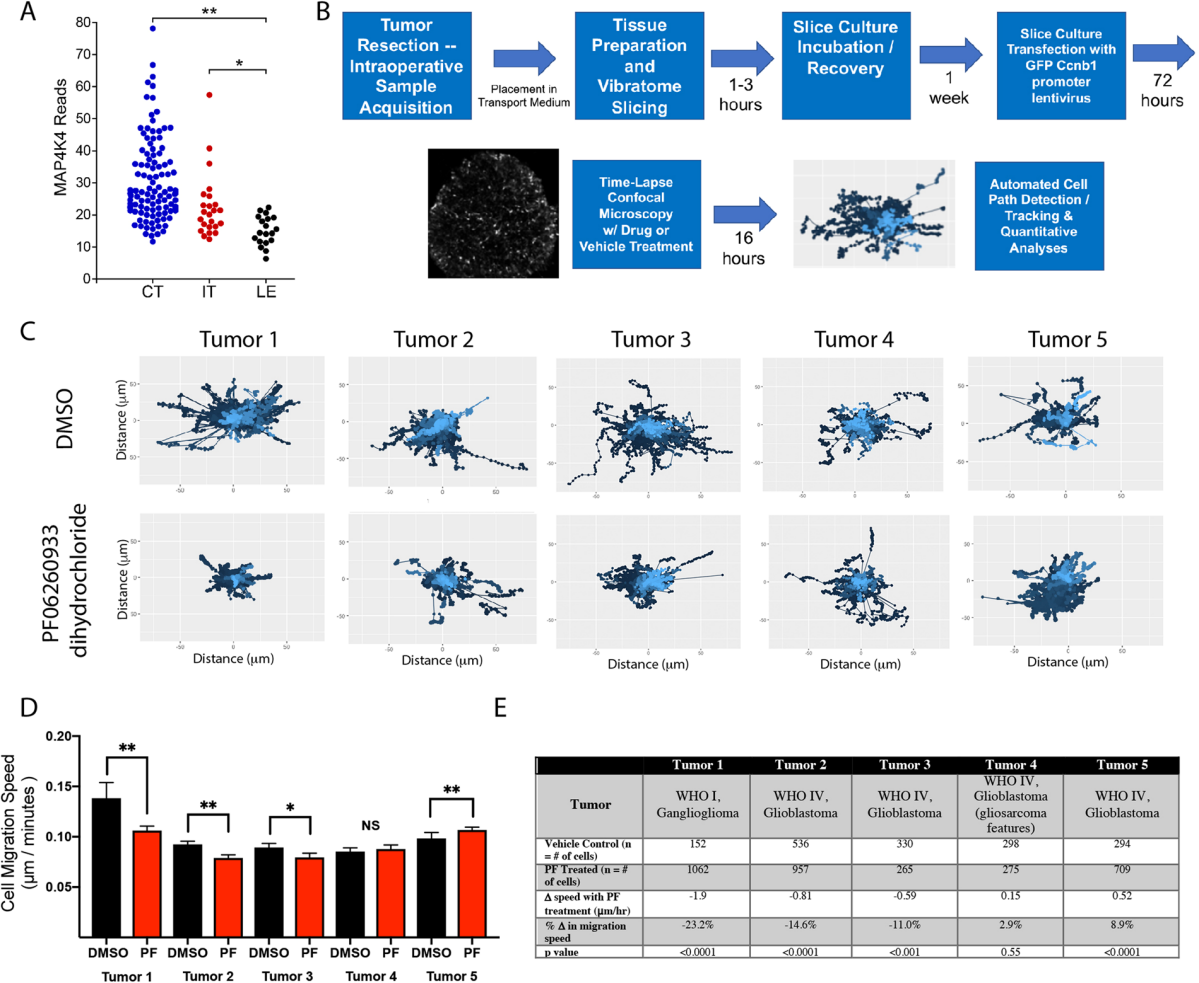
MAP4K inhibition decreases tumor cell migration in a subset of primary human glioma derived organotypic slice cultures. (**A**) MAP4K4 expression in tumor samples from the IVY Glioblastoma Atlas http://glioblastoma.alleninstitute.org/^16^. Each point corresponds to an individual laser-microdissected sample. Samples are organized by tumor sub-region, including cellular tumor (CT), infiltrating tumor (IT), and leading edge (LE), (*p < 0.002; **p < 6 × 10^−9^ rank sum test). No multiple-comparison corrections were applied. (**B**) Experimental workflow schematic for organotypic slice cultures outlining tissue acquisition, slicing, transfection, time-lapse imaging, and cell migration analyses. (**C**) Displacement maps from representative slice culture imaging regions from vehicle control (DMSO) top and PF06260933 dihydrochloride treated (bottom) for all 5 patient tumor samples highlight variability in baseline cell dispersion and efficacy of MAP4K4 inhibition. (**D**) Individual cell migration paths were detected by ADAPT and cell migration speed calculated using a distance over time method. Mean cell speeds from vehicle treated slice regions (black) and PF treated (red) for each tumor. Error bars represent 95% confidence interval (**p < 0.0001; *p < 0.001 Mann-Whitney). Individual cell migration speeds were aggregated from multiple tumor slices to capture population level effects of  MAP4K4 inhibition and groups were compared using the Mann-Whitney *U* Test given the non-normality of cell migration speed distributions. Significance was defined as p < 0.05. (**E**) Description of tumor pathology, number of cells tracked in the vehicle control and PF06260933 dihydrochloride treated regions and change in mean migration speed of the population.

To validate the necessity of MAP4K4 for invasion as well as migration, we returned to the transwell invasion assay. Two separate conditions were tested; the specific U138 MAP4K4 knockout line (Fig. 4A) and the U138 line using PF 06260933 dihydrochloride to inhibit MAP4K4 (Fig. 4B). We found that knocking-out MAP4K4 led to a 41% reduction in invasion compared to U138-Cas9 control (Fig. 4C, p < 0.05). Complete knock-out of MAP4K4 was confirmed by immunoblotting (Fig. 5A). Similarly, inhibiting MAP4K4 using 1 μM PF 06260933 dihydrochloride reduced invasion by 48% compared to U138 control (Fig. 4C, p < 0.05). Thus, specifically targeting MAP4K4 inhibits invasion, again validating the screen results.

### Intra- and inter-tumor heterogeneity in MAP4K4 expression in human gliomas

To investigate the distribution of MAP4K4 expression in human glioblastoma samples, we examined the IVY Glioblastoma Atlas (http://glioblastoma.alleninstitute.org/)^16^. RNA expression of MAP4K4 was heterogeneous across samples, and was elevated in the laser-dissected subregions from both the Cellular Tumor (CT, p < 6 × 10^−9^) and Infiltrating Tumor (IT, p < 0.002) relative to the Leading Edge, which consisted primarily of non-neoplastic cells at the tumor margins (Fig. 5A).

### Acute pharmacologic inhibition of MAP4K4 decreases tumor cell migration in a subset of human glioma organotypic slice cultures

The migration of human glioma cells is highly dependent on extracellular matrix and stromal cellular constituents, supported by engagement of different molecular machinery of migration in two versus three-dimensional tissue architectures^17^. *Ex vivo* organotypic slice culture models of human glioma tissue mimic the nature of the tumor microenvironment and allow for lentiviral cell labeling and time-lapse confocal microscopy (Fig. 5B)^18,19^. Intra- and inter-tumoral difference in human glioma cell migration utilizing quantitative path tracking techniques have been reported^18–20^. We sought to determine the efficacy of MAP4K4 inhibition in slice cultures generated from human tumor surgical resections. A total of 5 sets of slice cultures from unique patient tumors were prepared as previously described^18,19^. The tumor slices were treated with 1 μM of PF 06260933 dihydrochloride or vehicle control (1:1000 DMSO vol:vol) for 16 hours during concurrent imaging. All tumor tissue was collected from patients undergoing resection of suspected high-grade glioma from radiographic tumor appearance. From the cohort, 4 tumors were confirmed via clinical pathologic analysis to be glioblastoma (WHO IV) and one low grade tumor, a ganglioglioma (WHO I). Cell migration displacement maps were generated for vehicle control and PF 06260933 dihydrochloride treated tumor slices to visually assess for changes in cell population dispersion (Fig. 5C). Individual cell migration tracks across multiple tumor slices from unique micro-regions were combined and analyzed to represent the behavior of the aggregate tumor cell population. After 16 hours of PF treatment we found three tumors demonstrated a significant decrease in tumor cell migration speed ranging from −0.59 to −1.9 μm/hour (Fig. 5D, p < 0.001 to 0.0001). In the two remaining tumors there was a non-significant 0.15 μm/hour increase in migration speed (p = 0.55) and a significant 0.52 μm/hour increase (Fig. 5D, p < 0.0001). These data demonstrate heterogeneity in response to MAP4K4 inhibition, indicating the importance of discovering predictive biomarkers for the subset of tumors in which inhibition of this pathway effectively limits tumor cell migration.

### Glioblastoma cells transition to a non-invasive state in the absence of MAP4K4

**Figure 6:**
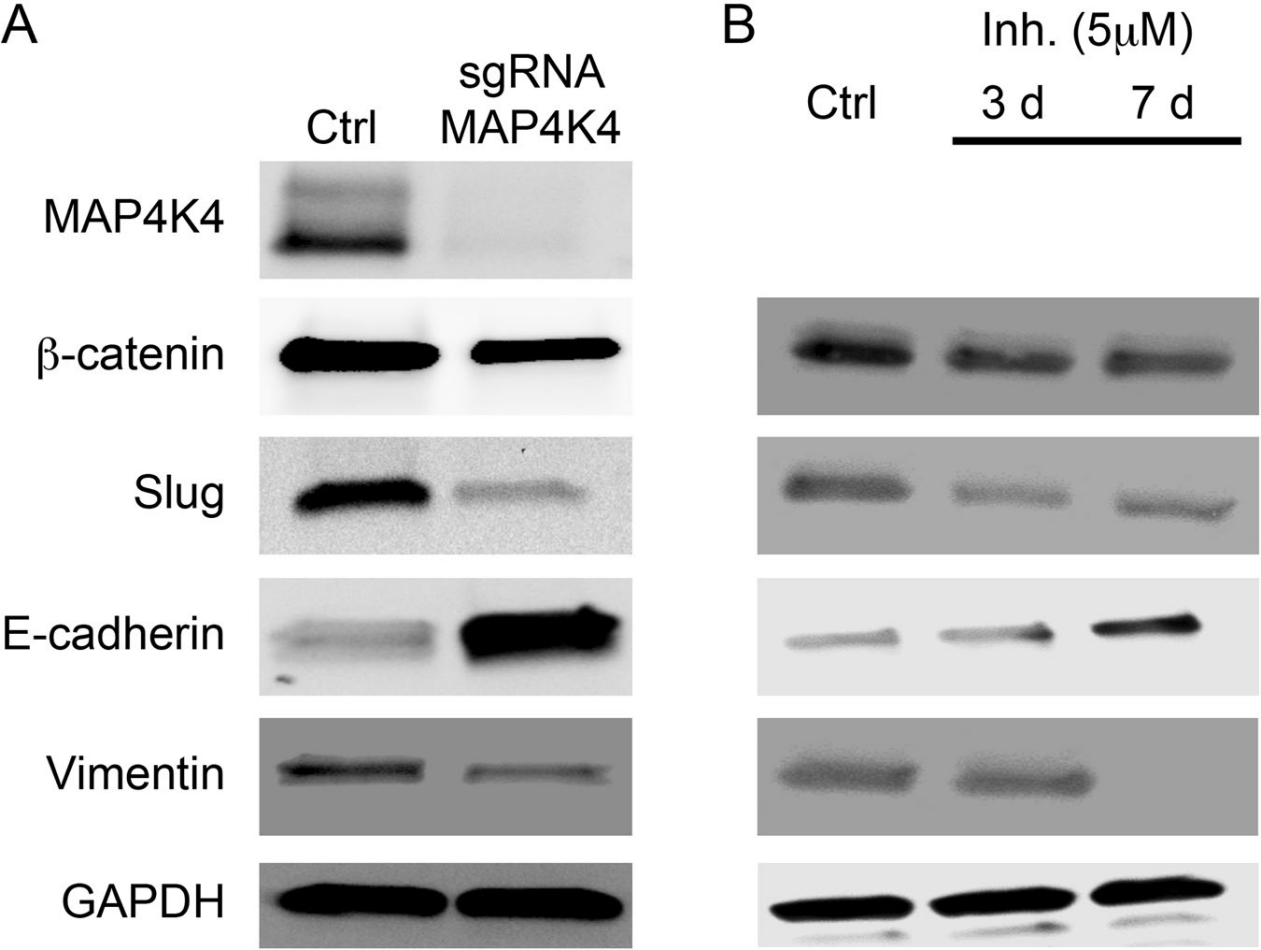
MAP4K4 regulates EMT in U138 cells. Immunoblots showing that either (**A**) knocking-out MAP4K4 or (**B**) inhibiting MAP4K4 leads to a protein expression profile more consistent with a non-invasive state. E-cadherin expression increases while β-catenin, Slug, and Vimentin decrease. GAPDH serves as a control. For MAP4K4 inhibitor (**B**) cells were cultured in 5 μM of PF06260933 dihydrochloride for either 3 or 7 days. Immunoblots were cropped. Full size immunoblots can be found in supplementary materials.

The epithelial to mesenchymal transition (EMT) is thought to underlie the transition of cancerous cells to a more invasive state. There are a number of pathways shown to regulate this transition. Perhaps most prominently, increased expression of three genetic markers slug, vimentin, and β-catenin is associated with a more invasive state. A decreased expression of the epithelial cell adhesion molecule, E-cadherin enhances the mesenchymal transition and also leads to a more invasive state^21^. We analyzed the expression of these markers in the MAP4K4 knockout line and found that loss of MAP4K4 resulted in a ~2-fold decrease in β-catenin and vimentin and a 3-fold decrease in slug, while E-cadherin had a 3.7-fold increase in protein expression compared to the control U138 cell line (Fig. 6A). These findings indicate that loss of MAP4K4 can inhibit the EMT transition thereby decreasing the invasive potential of the U138 cells. Furthermore, inhibition of MAP4K4 using PF 06260933 dihydrochloride also caused a decrease in the mesenchymal state through similar changes in the EMT markers. Longer treatments with PF06260933 dihydrochloride resulted in an even larger change in the EMT marker expression (Fig. 6B). Thus, loss of MAP4K4 function either by gene knockout or drug inhibition drives a transition of glioma cells towards a non-invasive state.

## Discussion

Identification of the essential genes for glioblastoma invasion is critical for developing molecularly targeted therapies and advancing treatment of these aggressive tumors. While a recent genome-wide CRISPR screen was performed to identify modulators of glioblastoma stem cell growth, survival and sensitivity to temozolomide^22^, here we report the first large-scale screen specifically designed for human glioblastoma invasion using CRISPR-Cas9 based gene disruption. We found a number of novel genes essential for glioblastoma invasion using a transwell invasion assay to separate phenotypes and validated the effects of selected candidates from our screen in an orthogonal migration assay and in an *ex vivo* human tumor organotypic slice assay. The general importance of each candidate gene for glioblastoma invasion was demonstrated. Our results identify MAP4K4 as a critical protein required for cell invasion and validate the necessity of MAP4K4 in four separate human glioblastoma cell lines, while also highlighting the utility of a specific drug inhibitor of MAP4K4. We found MAP4K4 expression correlates with epithelial-mesenchymal transition in glioblastoma and that eliminating MAP4K4 reverses this transition.

MAP4K4, a serine/threonine kinase that is expressed at highest levels in brain and testes^23^, is essential for normal development^24,25^, and has been implicated in immunity, cardiovascular disease, metabolic disease and cancer^26,27^. Several studies have supported the clinical significance of MAP4K4 in cancer. Northern blot RNA analysis demonstrates higher MAP4K4 expression in 67% of cancer cell lines from the National Cancer Institute compared to normal human tissue with the highest upregulation seen in glioblastoma samples (46.3 fold relative to normal brain)^23^. Furthermore, a negative association between MAP4K4 protein expression and patient prognosis has been made in a number of cancer types (colorectal, hepatocellular, pancreatic, lung and prostate)^28^. Notably, MAP4K4 expression is upregulated by EGFRvIII, the mutant EGFR receptor, expressed by glioblastomas that is constitutively active in the absence of ligand^29^. For these reasons, inhibition of MAP4K4 has been proposed as a treatment for the other common cancer types, and may have a therapeutic role in a wide variety of cancers. For example, MAP4K4 is required for maintenance of the malignant phenotype of lung adenocarcinoma including growth and metastatic potential^30^. One may speculate that treating patients who have cancer with high metastatic potential and upregulation of MAP4K4 with a MAP4K4 inhibitor to prevent metastasis while they wait for resection or radiation of the primary tumor would be of benefit. This novel treatment strategy could provide an efficient path toward improved survival. Downregulating MAP4K4 in pancreatic cancer cells inhibits proliferation and invasion and enhances chemosensitivity^31^. This study suggests a MAP4K4 inhibitor could potentially serve as a meaningful adjuvant for this chemotherapy-resistant fatal cancer. Similar arguments can be made for the use of a MAP4K4 inhibitor in prostate, ovarian and malignant melanoma as well^32^.

Previous studies have suggested the importance of MAP4K4 in migration. Recently, MAP4K4 expression was shown to be increased in 30% of primary medulloblastoma tumors, particularly in the metastatic SHH β subtype, and was shown to promote the migratory and invasive behavior of medulloblastoma tumor cells^33^. A small interfering RNA screen for modulators of tumor cell motility in an ovarian cancer line identified MAP4K4 as a pro-migratory kinase^32^. MAP4K4 was also identified in a separate study using a yeast two-hybrid screen to identify proteins associated with a pro-migratory protein, Pyk2^34^. The emergence of MAP4K4 as the top hit from our screen further supports the importance of this gene in invasion.

**Figure 7:**
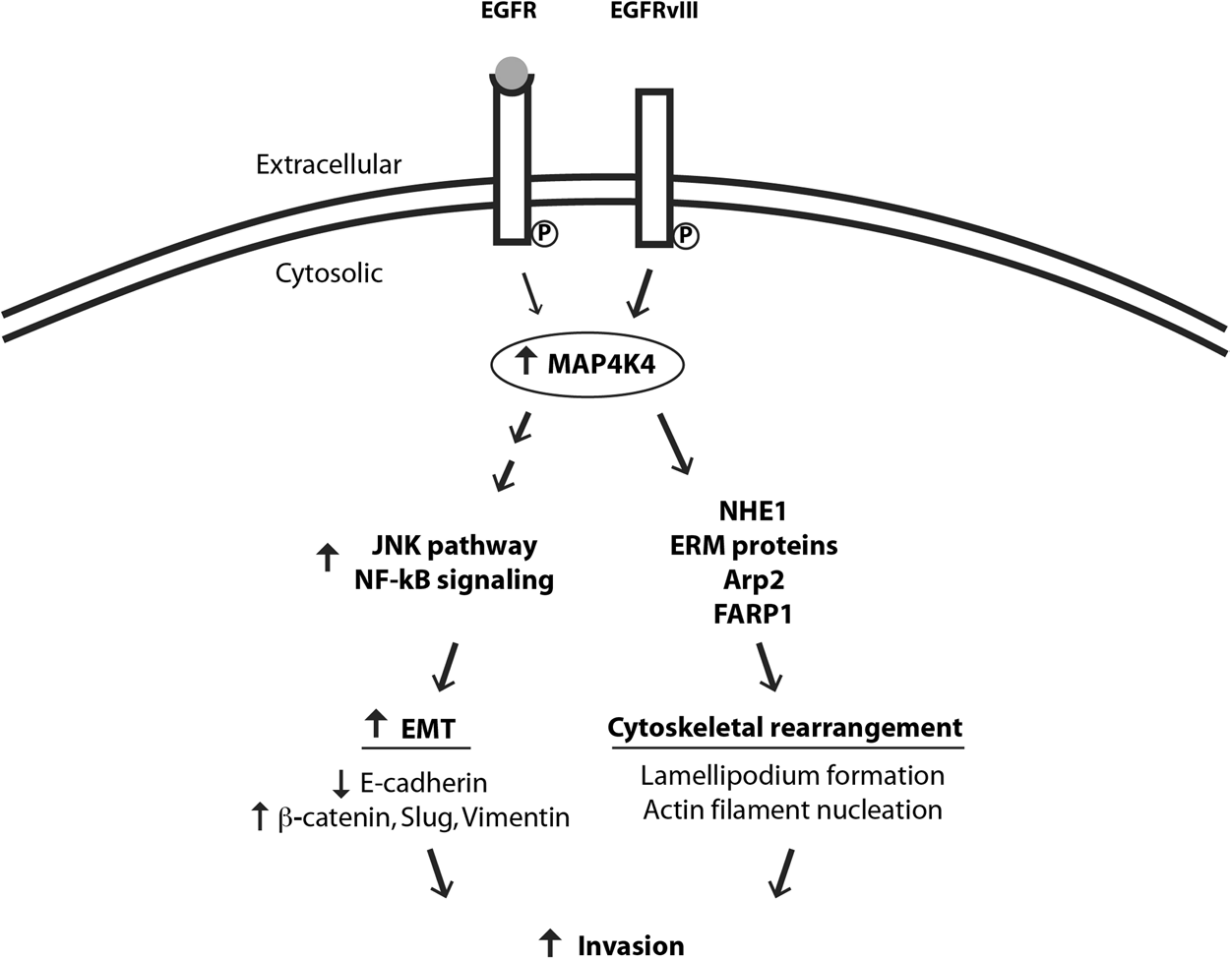
Schematic of a potential molecular mechanism by which MAP4K4 may promote invasion. Previous studies have shown EGFRvIII receptor, expressed at the GBM cell surface (double line), is constitutively active in the absence of ligand (gray circle) and upregulates MAP4K4 expression (thicker arrow) compared to the wild type EGFR^29^. Increased MAP4K4 activity in glioblastoma cells may promote migration and invasion by at least two mechanisms: JNK/NF-kB pathway activation (left) and phosphorylation of direct substrates (right). MAP4K4 activates the JNK/NF-kB pathways (left) to promote EMT as evidenced by lower protein expression of the cell-cell adhesion molecule, E-cadherin, and increased expression of β-catenin (a protein involved in linking cell adhesion molecules with gene transcription), slug (a transcription factor), and vimentin (an intermediate filament). Direct substrates for MAP4K4 phosphorylation (right) are NHE1, ERM proteins, Arp2, and FARP1. These proteins are essential for the cytoskeletal rearrangements underlying invasion.

EMT is the process by which cells transition from a noninvasive to an invasive state through activation of various pathways involved with turnover of cell adhesion molecules and remodeling of the cytoskeleton. We found MAP4K4 influences EMT markers in glioblastoma cells. Morphological changes in cells from a cobblestone-like resting state to a spindle shaped invasive state correlate with EMT and we observed these morphological changes in our MAP4K4 knock-out and control lines, respectively, as well (Fig. 4)^35^. In further support of the hypothesis that MAP4K4 influences EMT in glioblastoma cells, we found that high levels of MAP4K4 expression correlated with protein markers of an invasive state (β-catenin, slug and vimentin), and that inhibiting MAP4K4 caused cells to transition into a non-invasive state as defined by increased expression of E-cadherin (Figs 5–7).

The specific underlying signaling mechanisms through which MAP4K4 influences EMT and invasion are unknown. Several downstream pathways of MAP4K4 (MAPK/JNK, Notch, JAK-STAT, NF-kB) have been implicated in regulating tumorigenic properties of cells (migration, cell invasion, proliferation, apoptosis) in a context-dependent manner through both phosphorylation-dependent mechanisms and protein-protein interactions^28^. In hepatocellular carcinoma, MAP4K4 promotes EMT and invasiveness through activation of JNK and NF-kB signaling^35^. MAP4K4 may activate JNK directly or through other kinases. MAP4K4 exerts effects on the cytoskeleton through JNK-independent pathways as well.

Direct phosphorylation substrates of MAP4K4 include NHE1^36^, ERM proteins (ezrin, radixin, moesin)^25,37^, Arp2^38^ and FARP1^39^, providing an explanation on how MAP4K4 activity may promote cytoskeletal rearrangement (Fig. 7). Phosphorylation of ERM proteins and Arp2 provide a mechanism of MAP4K4-induced actin filament nucleation and lamellipodium formation in response EGFR activation. Notably, Arp2 (ACTR2) was one of our significant screen results and was also validated in the scratch assay. Thus, the identification of multiple gene hits from a single pathway is consistent with the central importance of this pathway in glioblastoma invasion. Future studies will be necessary to examine the EGFR/MAP4K4/Arp2 pathway in glioblastoma invasion further.

Given the multitude of downstream pathways MAP4K4 can potentially influence to promote invasion, inhibiting MAP4K4 may have widespread effects on cell physiology. However, we found MAP4K4 inhibition surprisingly had a specific effect on invasion and migration with little impact on cell survival. Promisingly, MAP4K4 inhibitors are actively being developed as an effective antidiabetic treatment^40,41^. While the specific MAP4K4 drug inhibitor PF 06260933 dihydrochloride (compound 16) used in our study has suitable pharmacokinetic properties in mouse, it unfortunately demonstrates time-dependent inhibition of the liver enzyme, CYP3A4, leading to drug accumulation and toxicity^40^. Newer compounds retain specificity for MAP4K4 while avoiding this unwanted side effect. However, each will need to be screened for central nervous system penetration^41^.

There are no clinically available pharmacologic agents that effectively limit glioblastoma invasion of surrounding healthy brain parenchyma. Human glioma organotypic slice cultures represent a patient-specific mechanism to test the efficacy of pre-clinical leads with anti-invasive activity. In this pilot assessment of MAP4K4 inhibition in 5 primary human gliomas, we found three tumor cell populations demonstrated a significant reduction of cell migration speed, one with no significant change, and one with a nominal significant enhanced migration with pharmacologic inhibition of MAP4K4. From our previous experience with human glioblastoma organotypic slices we have found significant intra and inter-tumoral heterogeneity in tumor cell migration^18,20^. This heterogeneity may limit the applicability of data from slice cultures generated from tissue representing an incomplete sampling tumor tissue to represent the tumor whole. However, we have attempted to limit the effect of this variability by aggregating cells tracked across multiple slice cultures in each condition. Future studies will focus on discovering patient-specific predictive biomarkers of MAP4K4 inhibition and synergistic pharmacologic combinations for limiting tumor cell migration.

In summary, we used a large-scale pooled library CRISPR-Cas9 approach to screen for genes necessary for glioblastoma invasion and discovered MAP4K4 as a promising therapeutic target. Future studies will focus on identifying the downstream molecular targets of MAP4K4 and determining whether MAP4K4 inhibition improves survival in orthotopic mouse brain tumor models *in vivo*.

## Materials and Methods

### Cell lines

U138, U87-MG, T98G and LN18 (American Type Culture Collection, Manassas, Virginia) cell lines were all cultured in Dulbecco’s modified Eagle medium (DMEM) supplemented with 10% fetal bovine serum (LN18 was cultured in 5% serum), 2 mM L-glutamine and 1% Penicillin-Streptomycin.

### Plasmids

The following plasmids were used in these studies: 1) SFFV-Cas9-BFP (vector backbone pMCB293, Cas9 protein fused with blue florescent protein), 2) puro-GFP (vector backbone pMCB306, puromycin resistance, green fluorescent protein and either a non-targeting sgRNA used as control or a sgRNA sequence targeting the specified gene), 3) trafficking/mitochondrial/motility and drug targets/kinase/phosphatase CRISPR/Cas9 deletion libraries (vector backbone pMCB320, puromycin resistance, mCherry protein and sgRNAs targeting 4,574 genes with 10 sgRNA sequences per gene). The following sequences were cloned into the puro-GFP plasmid to target the specified genes: MAP4K4 sequence 1 (TTGGCAGCCATCAAAGTTA), MAP4K4 sequence 2 (TTCTTCACAAGGTCTGTAA), ACTR2 (GTGGTGGTGTGCGACAACGGCAC), GGPS1 (ACAACTCAAAACTCCGACG), CKS1B (GCGTTCAGCAGAGTCA), NGLY1 (TGGACGGTGTGGCGAGT), CAP1 (ATCAGTAAAGAGATTGG), PPP1R8 (CCTGCTTCACTGTGTGTGG), EFTUD1 (GCTTGAAAACATCCGTC), PFN1 (ATGGCCGCGTCCTGAC), XPO1 (ATAAAAGTTGGCCAATGTT), ARPC3 (AAGCCATCTATTACTTCA).

### Lentivirus production

HEK293T cells were cultured in DMEM + 10% FBS + 1% Penicillin-Streptomycin and used to package lentivirus following a standard polyethylenimine (PEI) mediated method. Second generation packaging plasmids were used for SFFV-Cas9-BFP and third generation packaging plasmids for GFP control and the pooled library. After 72 hours, lentiviral containing media was harvested, filtered (0.45 μM pore, PVDF), and immediately used to infect the specified cell line.

### Generation of the CRISPR-Cas9 library in U138 cells

Lentiviral infection supplemented with 8 μg/ml polybrene was used to make a U138 line stably expressing SFFV-Cas9-BFP. U138 cells expressing BFP were sorted for using an ARIA-II (BD Biosciences, San Jose, CA) at the Stanford University FACS facility. The CRISPR-Cas9 deletion library line was made following established methods^12^. Briefly, lentivirus was used to infect 45 million U138 Cas9-BFP cells (1000 fold coverage) with the pooled library at an infection efficiency of ~20%. Infected cells underwent puromycin selection (1 μg/ml) and sgRNA expression was followed using flow cytometry. After 7 days of puromycin treatment, approximately 90% of cells were positive for mCherry.

### CRISPR screen using transwell invasion assay

U138 Cas9-BFP cells expressing the pooled libraries targeting 4574 genes and containing an additional 2,943 sgRNA negative controls were expanded for approximately 2 weeks *in vitro* at which point they were split into two groups. One group was used in the transwell invasion assay at 1000x coverage (each cell contains one sgRNA, 10 variable-length guides per gene, each sgRNA sequence represented 1000 fold) and the other group was harvested as a control. Sufficient library representation was confirmed by deep sequencing. Matrigel invasion chambers with 8-μm pore size were used to assess cell invasion. For the screen, 45 million cells expressing the pooled CRISPR library were seeded in serum free media in the upper chamber of a transwell at a density of 1 million cells per well in a 6-well format. Cells were allowed to invade toward 10% serum containing media in the lower chamber for 36 hours before cells in the upper and lower chamber were separately collected. Cells were first harvested from the bottom of the transwell using trypsin. The upper chamber was rinsed once with PBS to remove dead cells and cells which did not invade were extracted from the matrigel using dispase.

### Genomically integrated sgRNA analysis

Comparison of sgRNA enrichement in invasive, non-invasive and control populations of cells was achieved through recently published methods^12^. Genomic DNA was extracted from the 3 cell populations using a Qiagen Midi Kit and guides were isolated and amplified using PCR. Deep sequencing on an Illumina Nextseq was used to evaluate and sequence library composition. Guide composition was compared to the expected plasmid library using Cas9 high-Throughput maximum Likelihood Estimator (casTLE) version 1.0 (https://bitbucket.org/dmorgens/castle)^12,13^. casTLE is a statistical framework that determines the maximum effect size as well as a p-value with the associated effect. Genes were considered enriched in one population over another using a 10% False Discovery Rate cutoff.

### Pharamacological Transwell invasion assay to validate candidates

To validate candidates from the screen a 24 well format was used. Cells were plated at the same density as used in the screen (71,000 cells per well) and allowed to invade for 8 hours. For the MAP4K4 inhibitor condition, U138 cells were plated in serum free media containing 1 μM PF06260933 dihydrochloride in DMSO and DMSO was used as control. Noninvaded cells on the top of the insert were removed with a cotton swab. Invaded cells were fixed to the bottom of the insert with ethanol and stained with 0.1% cresyl violet. Three images were taken of each insert at 10x magnification using a light microscope with a Nikon camera attached, images were blinded and cells were manually counted using ImageJ software. p-values were calculated using an unpaired t-test for both conditions.

### Wound-healing assay

Cells were plated in a 24-well plate at a density of 50,000 (U87-MG), 75,000 (U138) or 150,000 (LN18 and T98G) cells per well such that there would be a confluent monolayer on the day after plating. At 24 hours after plating, two lines were scratched through the monolayer of cells using a 1000 μl pipette tip. The cells were rinsed and placed in an IncuCyte Live Cell Analysis Incubator at 37 °C and images were taken of the well every 30 mins for 24-72 hours. For drug inhibitor experiments, media containing the specified concentrations of PF06260933 dihydrochloride was added at the time of the scratch. DMSO was used as control.

### Wound-healing assay analysis

Scratch images were analyzed using an automated Matlab script (Matlab, Mathworks). Each image was processed using a sliding square area, 50 × 50 pixels in size, moving in 5 pixel increments across the image. The standard deviation of the pixels within the square area was calculated at each step as a measure of local structure in the image. Image regions containing high densities of cells (e.g. outside of the scratch area) were characterized by high levels of local image structure, while image regions containing few or no cells (e.g. inside the scratch are) were characterized by very low levels of local image structure. A histogram of standard deviation values for all local areas within the first image from each well was calculated. For each well, this histogram contained two distinct peaks – representing the set of local areas within the scratch and outside of the scratch, respectively. A threshold was set as the value halfway between these two peaks. For all subsequent images from that well, the fraction the image containing values less than this threshold (i.e. fraction of well containing cells) was calculated. By determining the image structure threshold separately for each well, this method automatically controlled for variations in plating density and image quality across wells and across experiments. p-values were calculated using a rank sum test after Bonferonni multiple comparisons correction.

### IVY glioblastoma atlas analysis

RNAseq data from the IVY Glioblastoma Atlas project was downloaded from the publicly available database (http://glioblastoma.alleninstitute.org/) and analyzed with a custom Python script. All code is available upon request. The database contains read counts for individual genes in each laser-microdissected sample, as well as information about the origin of the tumor, the patient, and the sub-region within the tumor. To generate the distributions of MAP4K4 gene expression plotted in Fig. 5A, each sample was weighted equally, and only tumor sub-region was considered. Additional information about the tumor samples and the generation of this data set are available in the original publication^16^.

### Human organotypic slice culture preparation, tumor cell labeling, and quantitative cell migration analysis

Tumor tissue was directly acquired from human glioma surgical resections obtained under a Inova Health System Institutional Review Board approved protocol at Inova Fairfax Medical Campus. Patients provided informed consent for the described research protocol. Samples were processed and cultures maintained in Neurobasal A media supplemented with B27 (Gibco), as previously described^19^. Briefly, 1 week after slice culture derivation, tumor cells were transduced with a lentiviral vector expressing green fluorescent protein (GFP) under the control of the human *Ccnb1* promoter. Approximately 3 days later, we assessed baseline migratory behavior using time-lapse laser scanning confocal microscopy. For each well at least three individual slices were imaged with z-stacks through 156 microns of tissue, acquired approximately every 16 minutes for 55 cycles. Inserts containing slice cultures were incubated with media containing 1uM PF-06260933 dihydrochloride or DMSO (1:1000 v:v) for approximately 16 hours. Experimental workflow was completed over 10-12 days after tissue acquisition. Slice cultures were continuously inspected for microbial contaminants in the media and culture media was exchanged every 48 hours. For analysis, Z-stacks were converted to maximum intensity projections and automated cell path track analysis was conducted using the ImageJ plugin ADAPT^42^. Using the technique as previously published the mean speed of each individual cell was calculated as distance traveled over time^18^. Each migration path was translated to a common origin of a Cartesian coordinate system to create dispersion maps (generated in R).

### Cell viability assay

CellTiter-Blue Cell Viability Assay (Promega) was used to assess cell survival. 5,000 cells were plated per well in a 96-well plate and metabolic activity as a measure of viability was measured at the specified time points over 48 h using 1:10 CellTiter-Blue measuring fluorescence at Ex: 544, Em: 590. The experiment was repeated at least three times. Data displayed as mean with standard error. ANOVA analysis was used to determine significance.

### Protein analysis

Protein extracts from total cells were harvested in RIPA buffer (0.15 M NaCl/0.05 mM Tris-HCl, pH 7.2/1% NP-40/1% sodium deoxycholate/0.1% SDS). Samples of 40 to 50 μg of total protein were separated by denaturing electrophoresis. The following antibodies were used for immunoblotting: rabbit anti-HGK/MAP4K4 (Cell Signaling, 3485), rabbit anti-vimentin (D21H3, Cell Signaling, 5741), rabbit anti-β-catenin (D10A8, Cell Signaling, 8480), rabbit anti-Slug (C19G7, Cell Signaling, 9585), rabbit anti-E-Cadherin (24E10, Cell Signaling, 3195), rabbit-anti-GAPDH (D16H11, Cell Signaling, 5174), anti-rabbit IgG HRP (Cell Signaling, 7074). Protein levels were determined by counting pixels using NIH Image.

## Supplementary information

Supplementary Figures

Supplementary Tables

## Data Availability

The authors agree to make materials, data and associated protocols promptly available to readers.

## References

[1] Mariani L (2001). Glioma cell motility is associated with reduced transcription of proapoptotic and proliferation genes: a cDNA microarray analysis. J Neurooncol.

[2] Lefranc F, Brotchi J, Kiss R (2005). Possible future issues in the treatment of glioblastomas: special emphasis on cell migration and the resistance of migrating glioblastoma cells to apoptosis. J Clin Oncol.

[3] Megalizzi V (2007). 4-IBP, a sigma1 receptor agonist, decreases the migration of human cancer cells, including glioblastoma cells, *in vitro* and sensitizes them *in vitro* and *in vivo* to cytotoxic insults of proapoptotic and proautophagic drugs. Neoplasia.

[4] Makrilia N, Kollias A, Manolopoulos L, Syrigos K (2009). Cell adhesion molecules: role and clinical significance in cancer. Cancer Invest.

[5] Friedl P, Wolf K (2003). Tumour-cell invasion and migration: diversity and escape mechanisms. Nat Rev Cancer.

[6] Jinek M (2012). A programmable dual-RNA-guided DNA endonuclease in adaptive bacterial immunity. Science.

[7] Wang T (2015). Identification and characterization of essential genes in the human genome. Science.

[8] Wang T, Wei JJ, Sabatini DM, Lander ES (2014). Genetic screens in human cells using the CRISPR-Cas9 system. Science.

[9] Tsai SQ (2015). GUIDE-seq enables genome-wide profiling of off-target cleavage by CRISPR-Cas nucleases. Nat Biotechnol.

[10] Gilbert LA (2014). Genome-Scale CRISPR-Mediated Control of Gene Repression and Activation. Cell.

[11] Mali P (2013). RNA-guided human genome engineering via Cas9. Science.

[12] Morgens DW (2017). Genome-scale measurement of off-target activity using Cas9 toxicity in high-throughput screens. Nat Commun.

[13] Morgens DW, Deans RM, Li A, Bassik MC (2016). Systematic comparison of CRISPR/Cas9 and RNAi screens for essential genes. Nat Biotechnol.

[14] Sciaccaluga M (2013). Functional cross talk between CXCR4 and PDGFR on glioblastoma cells is essential for migration. PLoS One.

[15] Nazarenko I (2012). PDGF and PDGF receptors in glioma. Ups J Med Sci.

[16] Puchalski RB (2018). An anatomic transcriptional atlas of human glioblastoma. Science.

[17] Beadle C (2008). The role of myosin II in glioma invasion of the brain. Mol Biol Cell.

[18] Parker JJ (2013). Gefitinib selectively inhibits tumor cell migration in EGFR-amplified human glioblastoma. Neuro Oncol.

[19] Parker, J. J., Lizarraga, M., Waziri, A. & Foshay, K. M. A Human Glioblastoma Organotypic Slice Culture Model for Study of Tumor Cell Migration and Patient-specific Effects of Anti-Invasive Drugs. *J Vis Exp*, 10.3791/53557 (2017). 10.3791/53557 PMC5612552 28784966

[20] Parker JJ (2018). Intratumoral heterogeneity of endogenous tumor cell invasive behavior in human glioblastoma. Sci Rep.

[21] Zeisberg M, Neilson EG (2009). Biomarkers for epithelial-mesenchymal transitions. J Clin Invest.

[22] MacLeod G (2019). Genome-Wide CRISPR-Cas9 Screens Expose Genetic Vulnerabilities and Mechanisms of Temozolomide Sensitivity in Glioblastoma Stem Cells. Cell Rep.

[23] Wright JH (2003). The STE20 kinase HGK is broadly expressed in human tumor cells and can modulate cellular transformation, invasion, and adhesion. Mol Cell Biol.

[24] Xue Y (2001). Mesodermal patterning defect in mice lacking the Ste20 NCK interacting kinase (NIK). Development.

[25] Vitorino P (2015). MAP4K4 regulates integrin-FERM binding to control endothelial cell motility. Nature.

[26] Chuang HC, Wang X, Tan TH (2016). MAP4K Family Kinases in Immunity and Inflammation. Adv Immunol.

[27] Virbasius JV, Czech MP (2016). Map4k4 Signaling Nodes in Metabolic and Cardiovascular Diseases. Trends Endocrinol Metab.

[28] Gao X, Gao C, Liu G, Hu J (2016). MAP4K4: an emerging therapeutic target in cancer. Cell Biosci.

[29] Ramnarain DB (2006). Differential gene expression analysis reveals generation of an autocrine loop by a mutant epidermal growth factor receptor in glioma cells. Cancer Res.

[30] Gao X (2017). MAP4K4 is a novel MAPK/ERK pathway regulator required for lung adenocarcinoma maintenance. Mol Oncol.

[31] Zhao G (2013). miRNA-141, downregulated in pancreatic cancer, inhibits cell proliferation and invasion by directly targeting MAP4K4. Mol Cancer Ther.

[32] Collins CS (2006). A small interfering RNA screen for modulators of tumor cell motility identifies MAP4K4 as a promigratory kinase. Proc Natl Acad Sci USA.

[33] Tripolitsioti D (2018). MAP4K4 controlled integrin beta1 activation and c-Met endocytosis are associated with invasive behavior of medulloblastoma cells. Oncotarget.

[34] Loftus JC (2013). A Novel Interaction between Pyk2 and MAP4K4 Is Integrated with Glioma Cell Migration. J Signal Transduct.

[35] Feng XJ (2016). MAP4K4 promotes epithelial-mesenchymal transition and metastasis in hepatocellular carcinoma. Tumour Biol.

[36] Yan W, Nehrke K, Choi J, Barber DL (2001). The Nck-interacting kinase (NIK) phosphorylates the Na+ − H+ exchanger NHE1 and regulates NHE1 activation by platelet-derived growth factor. J Biol Chem.

[37] Baumgartner M (2006). The Nck-interacting kinase phosphorylates ERM proteins for formation of lamellipodium by growth factors. Proc Natl Acad Sci USA.

[38] LeClaire LL, Rana M, Baumgartner M, Barber DL (2015). The Nck-interacting kinase NIK increases Arp2/3 complex activity by phosphorylating the Arp2 subunit. J Cell Biol.

[39] Schwaid AG (2015). MAP4K4 Is a Threonine Kinase That Phosphorylates FARP1. ACS Chem Biol.

[40] Ammirati M (2015). Discovery of an *in Vivo* Tool to Establish Proof-of-Concept for MAP4K4-Based Antidiabetic Treatment. ACS Med Chem Lett.

[41] Dow RL (2018). 2-Aminopyridine-Based Mitogen-Activated Protein Kinase Kinase Kinase Kinase 4 (MAP4K4) Inhibitors: Assessment of Mechanism-Based Safety. J Med Chem.

[42] Barry DJ, Durkin CH, Abella JV, Way M (2015). Open source software for quantification of cell migration, protrusions, and fluorescence intensities. J Cell Biol.

