# Supplementary material for: Targeted genomic CRISPR-Cas9 screen identifies MAP4K4 as essential for glioblastoma invasion
Source: Sci Rep. 2019 Sep 30;9:14020. doi: 10.1038/s41598-019-50160-w (PMC6768851; doi:10.1038/s41598-019-50160-w)

Title: Targeted genomic CRISPR-Cas9 screen identifies MAP4K4 as essential for glioblastoma invasion

Authors: Laura M Prolo[1], Amy Li[2], Scott F Owen[3], Jonathon J Parker[1], Kara Foshay[4], Ryan T Nitta[1], David W Morgens[2], Sara Bolin[1], Christy M Wilson[1], Johana C M Vega L[1], Emily J Luo[1], Gigi Nwagbo[1], Allen Waziri[4], Gordon Li[1], Richard J Reimer[5], Michael C Bassik[2], Gerald A Grant*[1]

[1]Stanford University School of Medicine, Department of Neurosurgery, 300 Pasteur Dr, Stanford, CA 94305

[2]Stanford University School of Medicine, Department of Genetics, 291 Campus Dr, Stanford, CA 94305

[3]J. David Gladstone Institute of Neurological Disease, 1650 Owens St, San Francisco, CA 94158

[4] Inova Neuroscience and Spine Institute, Inova Health Systems, 8110 Gatehouse Rd, Falls Church, VA, 22042

[5]Stanford University School of Medicine, Department of Neurology, 300 Pasteur Dr, Stanford, CA 94305

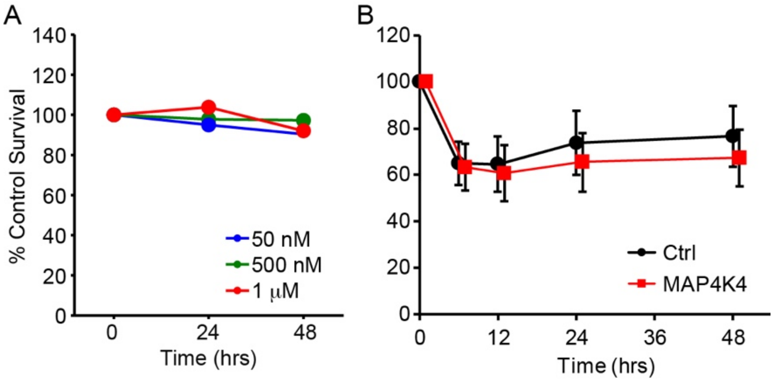

Supplementary Figure 1: U138 cell viability in the presence of MAP4K4 inhibitor or MAP4K4 knock-out. **(A)** Quantification of U138 cell viability in presence of PF06260933 dihydrochloride normalized to control over 48 hours. Data displayed as mean with standard error, n=4 for each drug concentration and time point. (B) Quantification of U138 Cas9-control (black) or U138 Cas9-sgMAP4K4 (red) viability normalized to t=0. Data are mean with standard error, n=12 for each time point. There was no significant difference in viability between control and MAP4K4 knock-out lines, $p>0.05$.

Full size immunoblots are presented below:

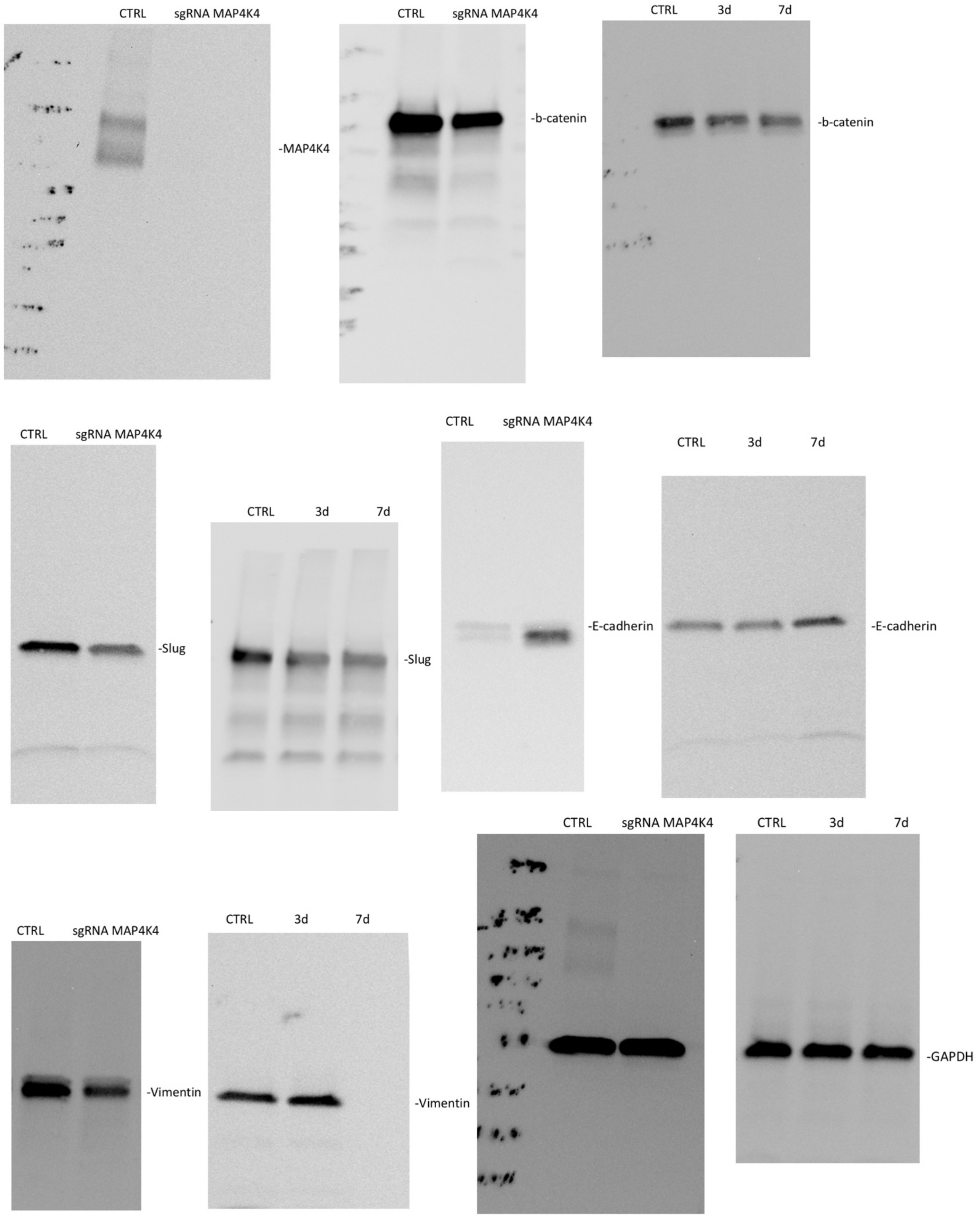

Supplement: Supplementary file 1 — Supplementary Figures [file 41598_2019_50160_MOESM1_ESM.pdf]
