# Supplementary material for: Targeted genomic CRISPR-Cas9 screen identifies MAP4K4 as essential for glioblastoma invasion
Source: Sci Rep. 2019 Sep 30;9:14020. doi: 10.1038/s41598-019-50160-w (PMC6768851; doi:10.1038/s41598-019-50160-w)
Supplement: Supplementary file 2 — Supplementary Tables [file 41598_2019_50160_MOESM2_ESM.pdf]

Title: Targeted genomic CRISPR-Cas9 screen identifies MAP4K4 as essential for glioblastoma invasion

Authors: Laura M Prolo[1], Amy Li[2], Scott F Owen[3], Jonathon J Parker[1], Kara Foshay[4], Ryan T Nitta[1], David W Morgens[2], Sara Bolin[1], Christy M Wilson[1], Johana C M Vega L[1], Emily J Luo[1], Gigi Nwagbo[1], Allen Waziri[4], Gordon Li[1], Richard J Reimer[5], Michael C Bassik[2], Gerald A Grant*[1]

[1]Stanford University School of Medicine, Department of Neurosurgery, 300 Pasteur Dr, Stanford, CA 94305

[2]Stanford University School of Medicine, Department of Genetics, 291 Campus Dr, Stanford, CA 94305

[3]J. David Gladstone Institute of Neurological Disease, 1650 Owens St, San Francisco, CA 94158

[4] Inova Neuroscience and Spine Institute, Inova Health Systems, 8110 Gatehouse Rd, Falls Church, VA, 22042

[5]Stanford University School of Medicine, Department of Neurology, 300 Pasteur Dr, Stanford, CA 94305

Supplementary Table 1: Genes targeted by sgRNAs disenriched from the library

<table>
<tr><th>Symbol</th><th>GeneInfo</th><th>Function</th></tr>
<tr><td>RAB4B</td><td>RAB4B, member RAS oncogene family</td><td>molecular_function|GTP binding|</td></tr>
<tr><td>PTPN20B</td><td>protein tyrosine phosphatase, non-receptor type 20B</td><td>protein tyrosine phosphatase activity|</td></tr>
<tr><td>NEDD8-MDP1</td><td>NEDD8-MDP1 readthrough</td><td>None</td></tr>
<tr><td>FDX1L</td><td>ferredoxin 1-like</td><td>electron carrier activity|metal ion binding|2 iron, 2 sulfur cluster binding|</td></tr>
<tr><td>GOSR2</td><td>golgi SNAP receptor complex member 2</td><td>transporter activity|</td></tr>
<tr><td>CDY2A</td><td>chromodomain protein, Y-linked, 2A</td><td>None</td></tr>
<tr><td>STX16</td><td>syntaxin 16</td><td>SNAP receptor activity|protein binding|</td></tr>
<tr><td>SLC25A10</td><td>solute carrier family 25 (mitochondrial carrier; dicarboxylate transporter), member 10</td><td>dicarboxylic acid transmembrane transporter activity|protein binding|</td></tr>
<tr><td>VKORC1</td><td>vitamin K epoxide reductase complex, subunit 1</td><td>vitamin-K-epoxide reductase (warfarin-sensitive) activity|quinone binding|</td></tr>
<tr><td>UGT1A8</td><td>UDP glucuronosyltransferase 1 family, polypeptide A8</td><td>retinoic acid binding|enzyme inhibitor activity|steroid binding|fatty acid binding|drug binding|glucuronosyltransferase activity|enzyme binding|protein homodimerization activity|protein homodimerization activity|protein heterodimerization activity|protein heterodimerization activity|</td></tr>
<tr><td>MRPL30</td><td>mitochondrial ribosomal protein L30</td><td>None</td></tr>
<tr><td>CDY1</td><td>chromodomain protein, Y-linked, 1</td><td>None</td></tr>
<tr><td>HOGA1</td><td>4-hydroxy-2-oxoglutarate aldolase 1</td><td>4-hydroxy-2-oxoglutarate aldolase activity|4-hydroxy-2-oxoglutarate aldolase activity|protein homodimerization activity|</td></tr>
<tr><td>DDX19B</td><td>DEAD (Asp-Glu-Ala-Asp) box polypeptide 19B</td><td>RNA binding|helicase activity|ATP binding|</td></tr>
<tr><td>SQRDL</td><td>sulfide quinone reductase-like (yeast)</td><td>sulfide:quinone oxidoreductase activity|</td></tr>
<tr><td>CCL3L1</td><td>chemokine (C-C motif) ligand 3-like 1</td><td>None</td></tr>
<tr><td>GATC</td><td>glutamyl-tRNA(Gln) amidotransferase, subunit C</td><td>ATP binding|glutaminyl-tRNA synthase (glutamine-hydrolyzing) activity|</td></tr>
<tr><td>PRY2</td><td>PTPN13-like, Y-linked 2</td><td>None</td></tr>
<tr><td>CSNK2B</td><td>casein kinase 2, beta polypeptide</td><td>protein serine/threonine kinase activity|receptor binding|protein binding|transcription factor binding|protein kinase regulator activity|protein domain specific binding|identical protein binding|metal ion binding|</td></tr>
<tr><td>PPAN</td><td>peter pan homolog (Drosophila)</td><td>poly(A) RNA binding|</td></tr>
<tr><td>TMX2</td><td>thioredoxin-related transmembrane protein 2</td><td>molecular_function|</td></tr>
<tr><td>BLOC1S5</td><td>biogenesis of lysosomal organelles complex-1, subunit 5, muted</td><td>protein binding|</td></tr>
<tr><td>ARL17A</td><td>ADP-ribosylation factor-like 17A</td><td>None</td></tr>
<tr><td>TIMM23</td><td>translocase of inner mitochondrial membrane 23 homolog (yeast)</td><td>protein binding|P-P-bond-hydrolysis-driven protein transmembrane transporter activity|</td></tr>
</table>

KPNB1	karyopherin (importin) beta 1	protein binding|nuclear localization sequence binding|zinc ion binding|Ran GTPase binding|protein transporter activity|enzyme binding|protein domain specific binding|poly(A) RNA binding|
TRAPPC5	trafficking protein particle complex 5	None
PSMA2	proteasome (prosome, macropain) subunit, alpha type, 2	threonine-type endopeptidase activity|protein binding|
SNRPD2	small nuclear ribonucleoprotein D2 polypeptide 16.5kDa	protein binding|poly(A) RNA binding|
TMEM141	transmembrane protein 141	None
HSPA5	heat shock 70kDa protein 5 (glucose-regulated protein, 78kDa)	glycoprotein binding|calcium ion binding|protein binding|ATP binding|ATPase activity|enzyme binding|protein domain specific binding|ubiquitin protein ligase binding|ribosome binding|unfolded protein binding|chaperone binding|misfolded protein binding|
AMY1A	amylase, alpha 1A (salivary)	None
BCKDHA	branched chain keto acid dehydrogenase E1, alpha polypeptide	alpha-ketoacid dehydrogenase activity|3-methyl-2-oxobutanoate dehydrogenase (2-methylpropanoyl-transferring) activity|protein binding|carboxy-lyase activity|metal ion binding|
NDUFC2	NADH dehydrogenase (ubiquinone) 1, subcomplex unknown, 2, 14.5kDa	NADH dehydrogenase (ubiquinone) activity|
PTGR2	prostaglandin reductase 2	13-prostaglandin reductase activity|15-oxoprostaglandin 13-oxidase activity|
HMGCS1	3-hydroxy-3-methylglutaryl-CoA synthase 1 (soluble)	hydroxymethylglutaryl-CoA synthase activity|drug binding|isomerase activity|protein homodimerization activity|organic acid binding|
IFNAR2	interferon (alpha, beta and omega) receptor 2	type I interferon receptor activity|protein binding|protein kinase binding|type I interferon binding|
RNASET2	ribonuclease T2	RNA binding|ribonuclease activity|ribonuclease T2 activity|
DMPK	dystrophia myotonica-protein kinase	protein serine/threonine kinase activity|protein binding|ATP binding|myosin phosphatase regulator activity|heat shock protein binding|metal ion binding|
COG8	component of oligomeric golgi complex 8	None
BCO2	beta-carotene oxygenase 2	oxidoreductase activity, acting on single donors with incorporation of molecular oxygen, incorporation of two atoms of oxygen|metal ion binding|
NAA25	N(alpha)-acetyltransferase 25, NatB auxiliary subunit	None
DDT	D-dopachrome tautomerase	dopachrome isomerase activity|D-dopachrome decarboxylase activity|
GSTT2	glutathione S-transferase theta 2	glutathione transferase activity|
CSNK1G1	casein kinase 1, gamma 1	protein serine/threonine kinase activity|ATP binding|
GSK3A	glycogen synthase kinase 3 alpha	protein serine/threonine kinase activity|protein binding|ATP binding|protein kinase A catalytic subunit binding|tau-protein kinase activity|
NHP2L1	NHP2 non-histone chromosome protein 2-like 1 (S. cerevisiae)	RNA binding|protein binding|snoRNA binding|poly(A) RNA binding|
HMGCR	3-hydroxy-3-methylglutaryl-CoA reductase	hydroxymethylglutaryl-CoA reductase (NADPH) activity|protein binding|hydroxymethylglutaryl-CoA

		reductase activity|protein homodimerization activity|coenzyme binding|NADPH binding|
TNNI3K	TNNI3 interacting kinase	protein kinase activity|protein serine/threonine kinase activity|protein binding|ATP binding|protein C-terminus binding|troponin I binding|metal ion binding|
PPP3R1	protein phosphatase 3, regulatory subunit B, alpha	calcium-dependent protein serine/threonine phosphatase activity|calcium ion binding|protein binding|calmodulin binding|protein domain specific binding|
PPP1CB	protein phosphatase 1, catalytic subunit, beta isozyme	protein binding|phosphatase activity|myosin phosphatase activity|protein kinase binding|metal ion binding|myosin-light-chain-phosphatase activity|
AARS	alanyl-tRNA synthetase	tRNA binding|aminoacyl-tRNA editing activity|alanine-tRNA ligase activity|ATP binding|zinc ion binding|amino acid binding|
S100A7	S100 calcium binding protein A7	calcium ion binding|protein binding|zinc ion binding|zinc ion binding|RAGE receptor binding|
GBF1	golgi brefeldin A resistant guanine nucleotide exchange factor 1	ARF guanyl-nucleotide exchange factor activity|protein binding|
EIF4A3	eukaryotic translation initiation factor 4A3	mRNA binding|ATP-dependent RNA helicase activity|protein binding|ATP binding|poly(A) binding|poly(A) RNA binding|
SF3B1	splicing factor 3b, subunit 1, 155kDa	chromatin binding|protein binding|poly(A) RNA binding|
MTHFS	5,10-methenyltetrahydrofolate synthetase (5-formyltetrahydrofolate cyclo-ligase)	ATP binding|folic acid binding|5-formyltetrahydrofolate cyclo-ligase activity|5-formyltetrahydrofolate cyclo-ligase activity|
SDHAF2	succinate dehydrogenase complex assembly factor 2	protein binding|
BANF1	barrier to autointegration factor 1	DNA binding|protein binding|
UBC	ubiquitin C	protease binding|protein binding|poly(A) RNA binding|
RNGTT	RNA guanylyltransferase and 5'-phosphatase	mRNA guanylyltransferase activity|polynucleotide 5'-phosphatase activity|protein tyrosine phosphatase activity|GTP binding|protein tyrosine/serine/threonine phosphatase activity|RNA guanylyltransferase activity|triphosphatase activity|
PLK1	polo-like kinase 1	protein kinase activity|protein serine/threonine kinase activity|protein serine/threonine kinase activity|protein serine/threonine kinase activity|protein binding|ATP binding|microtubule binding|anaphase-promoting complex binding|kinase activity|protein kinase binding|
PPP1R10	protein phosphatase 1, regulatory subunit 10	DNA binding|protein phosphatase inhibitor activity|poly(A) RNA binding|metal ion binding|
CDC7	cell division cycle 7	protein kinase activity|protein serine/threonine kinase activity|protein binding|ATP binding|kinase activity|metal ion binding|
TXNRD1	thioredoxin reductase 1	thioredoxin-disulfide reductase activity|protein binding|electron carrier activity|protein disulfide oxidoreductase activity|flavin adenine dinucleotide binding|NADP binding|

MTAP	methylthioadenosine phosphorylase	phosphorylase activity|S-methyl-5-thioadenosine phosphorylase activity|
PTPN1	protein tyrosine phosphatase, non-receptor type 1	protein tyrosine phosphatase activity|protein tyrosine phosphatase activity|insulin receptor binding|protein binding|zinc ion binding|enzyme binding|protein kinase binding|receptor tyrosine kinase binding|poly(A) RNA binding|ephrin receptor binding|
SLC25A22	solute carrier family 25 (mitochondrial carrier: glutamate), member 22	L-glutamate transmembrane transporter activity|symporter activity|
SERPINB2	serpin peptidase inhibitor, clade B (ovalbumin), member 2	serine-type endopeptidase inhibitor activity|
SARS	seryl-tRNA synthetase	RNA binding|serine-tRNA ligase activity|ATP binding|
PPIA	peptidylprolyl isomerase A (cyclophilin A)	peptidyl-prolyl cis-trans isomerase activity|protein binding|peptide binding|poly(A) RNA binding|virion binding|unfolded protein binding|
LSM6	LSM6 homolog, U6 small nuclear RNA associated (S. cerevisiae)	protein binding|poly(A) RNA binding|
HGS	hepatocyte growth factor-regulated tyrosine kinase substrate	protein binding|protein domain specific binding|metal ion binding|
PTPN23	protein tyrosine phosphatase, non-receptor type 23	protein tyrosine phosphatase activity|protein binding|
SNRPD3	small nuclear ribonucleoprotein D3 polypeptide 18kDa	protein binding|enzyme binding|poly(A) RNA binding|histone pre-mRNA DCP binding|
CKS1B	CDC28 protein kinase regulatory subunit 1B	protein binding|cyclin-dependent protein serine/threonine kinase regulator activity|
VPS28	vacuolar protein sorting 28 homolog (S. cerevisiae)	protein binding|
XRN2	5'-3' exoribonuclease 2	nuclease activity|5'-3' exoribonuclease activity|zinc ion binding|5'-3' exonuclease activity|poly(A) RNA binding|
DCTN5	dynactin 5 (p25)	None
AURKA	aurora kinase A	protein kinase activity|protein serine/threonine kinase activity|protein serine/threonine/tyrosine kinase activity|protein binding|ATP binding|protein kinase binding|ubiquitin protein ligase binding|
NUP188	nucleoporin 188kDa	None
THG1L	tRNA-histidine guanylyltransferase 1-like (S. cerevisiae)	tRNA binding|magnesium ion binding|ATP binding|GTP binding|tRNA guanylyltransferase activity|tRNA guanylyltransferase activity|identical protein binding|
RAD51C	RAD51 paralog C	four-way junction DNA binding|DNA binding|protein binding|ATP binding|DNA-dependent ATPase activity|crossover junction endodeoxyribonuclease activity|
RAPGEF1	Rap guanine nucleotide exchange factor (GEF) 1	protein binding|Rap guanyl-nucleotide exchange factor activity|Rap guanyl-nucleotide exchange factor activity|SH3 domain binding|
DCTN6	dynactin 6	dynein binding|
TOMM20	translocase of outer mitochondrial membrane 20 homolog (yeast)	protein binding|P-P-bond-hydrolysis-driven protein transmembrane transporter activity|unfolded protein binding|

VCX3A	variable charge, X-linked 3A	None
FCF1	FCF1 rRNA-processing protein	poly(A) RNA binding|
DYNLRB1	dynein, light chain, roadblock-type 1	microtubule motor activity|identical protein binding|
ACTR10	actin-related protein 10 homolog (S. cerevisiae)	None
TMED2	transmembrane emp24 domain trafficking protein 2	protein binding|
ARHGAP8	Rho GTPase activating protein 8	Rho GTPase activator activity|protein binding|
PTPN11	protein tyrosine phosphatase, non-receptor type 11	phosphoprotein phosphatase activity|protein tyrosine phosphatase activity|protein tyrosine phosphatase activity|non-membrane spanning protein tyrosine phosphatase activity|non-membrane spanning protein tyrosine phosphatase activity|SH3/SH2 adaptor activity|insulin receptor binding|protein binding|protein domain specific binding|receptor tyrosine kinase binding|D1 dopamine receptor binding|phospholipase binding|insulin receptor substrate binding|peptide hormone receptor binding|
PPP1R2	protein phosphatase 1, regulatory (inhibitor) subunit 2	protein serine/threonine phosphatase inhibitor activity|protein binding|
TPSB2	tryptase beta 2 (gene/pseudogene)	serine-type endopeptidase activity|serine-type peptidase activity|
DHX37	DEAH (Asp-Glu-Ala-His) box polypeptide 37	nucleic acid binding|helicase activity|ATP binding|
ALG1	ALG1, chitobiosyldiphosphodolichol beta-mannosyltransferase	mannosyltransferase activity|chitobiosyldiphosphodolichol beta-mannosyltransferase activity|
GOLGA6L1	golgin A6 family-like 1	None
S100G	S100 calcium binding protein G	vitamin D binding|calcium ion binding|

Supplementary Table 2: Genes targeted by sgRNAs enriched in the non-invasive population

Symbol	GeneInfo	Function
MAP4K4	mitogen-activated protein kinase kinase kinase kinase 4	protein serine/threonine kinase activity|small GTPase regulator activity|protein binding|ATP binding|
AARS	alanyl-tRNA synthetase	tRNA binding|aminoacyl-tRNA editing activity|alanine-tRNA ligase activity|ATP binding|zinc ion binding|amino acid binding|
NARS	asparaginyl-tRNA synthetase	nucleic acid binding|asparagine-tRNA ligase activity|ATP binding|
ACTR2	ARP2 actin-related protein 2 homolog (yeast)	actin binding|ATP binding|
ABCE1	ATP-binding cassette, sub-family E (OABP), member 1	ATP binding|ribonuclease inhibitor activity|ATPase activity|iron-sulfur cluster binding|
TAOK1	TAO kinase 1	protein kinase activity|protein serine/threonine kinase activity|protein serine/threonine kinase activity|protein binding|ATP binding|kinase activity|transferase activity|
GAPDH	glyceraldehyde-3-phosphate dehydrogenase	glyceraldehyde-3-phosphate dehydrogenase (NAD+) (phosphorylating) activity|glyceraldehyde-3-phosphate

		dehydrogenase (NAD+) (phosphorylating) activity|protein binding|microtubule binding|peptidyl-cysteine S-nitrosylase activity|identical protein binding|NADP binding|NAD binding|
GGPS1	geranylgeranyl diphosphate synthase 1	dimethylallyltranstransferase activity|farnesyltranstransferase activity|geranyltranstransferase activity|metal ion binding|
CKS1B	CDC28 protein kinase regulatory subunit 1B	protein binding|cyclin-dependent protein serine/threonine kinase regulator activity|
SEH1L	SEH1-like (S. cerevisiae)	None
CDK9	cyclin-dependent kinase 9	DNA binding|chromatin binding|protein kinase activity|cyclin-dependent protein serine/threonine kinase activity|protein binding|ATP binding|RNA polymerase II carboxy-terminal domain kinase activity|snRNA binding|transcription regulatory region DNA binding|
EEF2	eukaryotic translation elongation factor 2	translation elongation factor activity|GTPase activity|GTP binding|translation activator activity|protein kinase binding|poly(A) RNA binding|
CDK1	cyclin-dependent kinase 1	protein kinase activity|protein serine/threonine kinase activity|cyclin-dependent protein serine/threonine kinase activity|cyclin-dependent protein serine/threonine kinase activity|protein binding|ATP binding|RNA polymerase II carboxy-terminal domain kinase activity|cyclin binding|Hsp70 protein binding|histone kinase activity|
RBMXL1	RNA binding motif protein, X-linked-like 1	nucleotide binding|RNA binding|
DHX30	DEAH (Asp-Glu-Ala-His) box helicase 30	chromatin binding|double-stranded RNA binding|protein binding|ATP binding|ATP-dependent helicase activity|poly(A) RNA binding|
NGLY1	N-glycanase 1	peptide-N4-(N-acetyl-beta-glucosaminyl)asparagine amidase activity|protein binding|metal ion binding|
CAP1	CAP, adenylate cyclase-associated protein 1 (yeast)	actin binding|
HARS	histidyl-tRNA synthetase	histidine-tRNA ligase activity|ATP binding|
PPP1R8	protein phosphatase 1, regulatory subunit 8	DNA binding|RNA binding|endonuclease activity|protein serine/threonine phosphatase inhibitor activity|protein binding|protein phosphatase type 1 regulator activity|ribonuclease E activity|
EFTUD1	elongation factor Tu GTP binding domain containing 1	translation elongation factor activity|GTPase activity|GTP binding|ribosome binding|
TRAPPC3	trafficking protein particle complex 3	protein binding|
PDGFRA	platelet-derived growth factor receptor, alpha polypeptide	transmembrane receptor protein tyrosine kinase activity|platelet-derived growth factor alpha-receptor activity|platelet-derived growth factor alpha-receptor activity|vascular endothelial growth factor-activated receptor activity|platelet-derived growth factor receptor binding|protein binding|ATP binding|vascular endothelial growth factor binding|protein homodimerization activity|platelet-derived growth factor binding|platelet-derived growth factor binding|
PFN1	profilin 1	adenyl-nucleotide exchange factor activity|actin binding|receptor binding|protein binding|phosphatidylinositol-4,5-bisphosphate binding|Rho GTPase binding|poly(A) RNA binding|proline-rich region binding|

FCGR1B	Fc fragment of IgG, high affinity Ib, receptor (CD64)	immunoglobulin receptor activity|IgG binding|
MRPL43	mitochondrial ribosomal protein L43	structural constituent of ribosome|poly(A) RNA binding|
XPO1	exportin 1	RNA binding|transporter activity|nucleocytoplasmic transporter activity|protein binding|Ran GTPase binding|protein transporter activity|protein domain specific binding|
ABCF1	ATP-binding cassette, sub-family F (GCN20), member 1	protein binding|ATP binding|ATP binding|translation factor activity, nucleic acid binding|translation activator activity|ATPase activity|ribosome binding|poly(A) RNA binding|
BTAF1	BTAF1 RNA polymerase II, B-TFIID transcription factor-associated, 170kDa	DNA binding|sequence-specific DNA binding transcription factor activity|helicase activity|ATP binding|
YARS	tyrosyl-tRNA synthetase	tRNA binding|tyrosine-tRNA ligase activity|signal transducer activity|interleukin-8 receptor binding|ATP binding|poly(A) RNA binding|
NSF	N-ethylmaleimide-sensitive factor	protein binding|ATP binding|syntaxin-1 binding|protein kinase binding|PDZ domain binding|protein complex binding|ATPase activity, coupled|metal ion binding|
NHP2L1	NHP2 non-histone chromosome protein 2-like 1 (S. cerevisiae)	RNA binding|protein binding|snoRNA binding|poly(A) RNA binding|
ARPC3	actin related protein 2/3 complex, subunit 3, 21kDa	actin binding|structural constituent of cytoskeleton|protein binding|
LSM6	LSM6 homolog, U6 small nuclear RNA associated (S. cerevisiae)	protein binding|poly(A) RNA binding|
